# Implementation of a Lightweight Semantic Segmentation Algorithm in Road Obstacle Detection

**DOI:** 10.3390/s20247089

**Published:** 2020-12-10

**Authors:** Bushi Liu, Yongbo Lv, Yang Gu, Wanjun Lv

**Affiliations:** School of Traffic and Transportation, Beijing Jiaotong University, Beijing 100044, China; 17114257@bjtu.edu.cn (B.L.); 18114004@bjtu.edu.cn (Y.G.); 18114022@bjtu.edu.cn (W.L.)

**Keywords:** road obstacle detection, deep learning, semantic segmentation, spatial information network, loss function

## Abstract

Due to deep learning’s accurate cognition of the street environment, the convolutional neural network has achieved dramatic development in the application of street scenes. Considering the needs of autonomous driving and assisted driving, in a general way, computer vision technology is used to find obstacles to avoid collisions, which has made semantic segmentation a research priority in recent years. However, semantic segmentation has been constantly facing new challenges for quite a long time. Complex network depth information, large datasets, real-time requirements, etc., are typical problems that need to be solved urgently in the realization of autonomous driving technology. In order to address these problems, we propose an improved lightweight real-time semantic segmentation network, which is based on an efficient image cascading network (ICNet) architecture, using multi-scale branches and a cascaded feature fusion unit to extract rich multi-level features. In this paper, a spatial information network is designed to transmit more prior knowledge of spatial location and edge information. During the course of the training phase, we append an external loss function to enhance the learning process of the deep learning network system as well. This lightweight network can quickly perceive obstacles and detect roads in the drivable area from images to satisfy autonomous driving characteristics. The proposed model shows substantial performance on the Cityscapes dataset. With the premise of ensuring real-time performance, several sets of experimental comparisons illustrate that SP-ICNet enhances the accuracy of road obstacle detection and provides nearly ideal prediction outputs. Compared to the current popular semantic segmentation network, this study also demonstrates the effectiveness of our lightweight network for road obstacle detection in autonomous driving.

## 1. Introduction

Nowadays, autonomous driving has become a research hotspot in the field of intelligent transportation. As one of the significant computer vision designs, the main task of deep learning in autonomous driving systems is to assist the car to sense the surrounding environment in time during the driving process. Through the detection, recognition, and tracking of target objects, obstacles such as pedestrians and vehicles can be avoided, thereby improving the safety of car driving. In this regard, researchers have conducted plenty of research on image classification and detection using deep convolutional neural networks, such as scene parsing [1], pose estimation [2], object detection [3], and collision avoidance [4]. Semantic segmentation [5] is a major branch of deep learning. Its main goal is to predict the label for each pixel in the image, so as to mine deep feature information and obtain accurate detection results, which is of great significance for autonomous driving.

Recently, semantic segmentation has been very popular in a range of environment perception tasks. Wong [6] proposed a feedback-based deep semantic segmentation which can incorporate the spatial context through appending an output feedback mechanism without a post-process step such as conditional random field (CRF) refinement. Junaid [7] proposed a multi-feature view-based shallow convolutional neural network (MVS-CNN), which utilizes the abstract features extracted from the gradient information of the image, improving the semantic segmentation of the road region. In order to capture and transmit the road-specific contextual information, research has been focused on the spatial information inference structures (SIISs), which can learn both the local visual characteristics of the road and the global spatial structure information [8]. In order to accurately extract linear features, a novel dilated convolution, which contains vertical and horizontal kernels (DVH), was introduced into the feature extraction task of a semantic segmentation network [9]. Mobile laser scanning (MLS) technology has also been widely used in road image segmentation and recognition [10]. Balado, et al. [11] used the point cloud acquired by the sensor to apply PointNet to scene segmentation and performed semantic segmentation on the main targets of the road environment to understand real-time road conditions. Semantic segmentation can also be enhanced by wavelet transform, where the symmetric fully convolutional neural network is designed to carry out lane-marking segmentation [12]. However, in actual application scenarios, autonomous driving has extremely strict requirements for real-time road and obstacle detection methods. It is desirable to develop novel semantic segmentation models assuring a fast inference speed for autonomous vehicles.

In order to solve this problem, a multitude of scholars have constructed real-time semantic segmentation models. Sun [13] proposed a real-time fusion semantic segmentation network called RFNet, which can effectively use complementary cross-mode information to conduct real-time RGB-D fusion semantic segmentation research, enriching the unforeseen hazard identification in real scenes. Zhao [14] proposed an image cascade network (ICNet) based on the pyramid scene analysis network, which integrates medium- and high-resolution features, while taking into account the segmentation accuracy, and uses the cascade strategy to accelerate the realization of real-time image semantic segmentation. By directly connecting the shallow feature map in the encoding module to the decoding module of the corresponding size, LinkNet not only uses the accurate position information of the shallow layer, but also does not increase the redundant parameters and calculations, so the calculation speed is improved under the premise of ensuring the accuracy [15]. The ENet network is designed with an asymmetrical codec structure. The convolution operation is decomposed by low-rank approximation, which can ensure the accuracy of segmentation while significantly reducing the amount of calculation. It is a real-time segmentation network that can complete tasks such as pixel labeling and scene analysis [16]. In addition, the lightweight network LEDNet proposed by Wang, et al. [17] develops the residual module based on the ReNet network encoder and introduces an attention mechanism in the decoder to predict the semantic label of each pixel. Thus, while enhancing the feature expression ability, it also reduces the amount of network calculation. Therefore, we can conclude that for the purpose of improving the inference speed of the semantic segmentation network, it is general to design novel semantic segmentation models, compression models, or advanced modules to meet real-time requirements. The key point to realize real-time semantic segmentation is to increase the segmentation speed while ensuring the accuracy of segmentation. Hence, balancing the segmentation accuracy and inference speed of the model will be one of the crucial research directions in the future.

Although the semantic segmentation network has excellent performance, especially in the parsing task of outdoor road scenes, the problem of rough segmentation of target edges cannot be ignored. The authors of [18] used a new kind of upsampling method to optimize the image segmentation of the edge of the object, which more comprehensively retains the edge information of the detection target and obtains a better boundary mask. Mostajabi, et al. [19] used superpixels as the basic unit to extract image features, and then input the features into the VGG16 network [20]. This method transforms the pixel-level semantic segmentation problem into a classification problem based on superpixels. By combining the spatial context information of superpixels, the extracted image features take into account both local and global information. Similarly, when CRF is used as the subsequent optimization process, the higher order potential (HOP) based on superpixels is increased and embedded in the CNN for end-to-end training to upgrade the accuracy of image semantic segmentation [21]. Feng, et al. [22] employed a boundary-enhanced loss (BEL) for learning exquisite boundaries-based salient object detection methods.

In this paper, considering the performance and speed in the environment perception and road obstacle detection, an ICNet is employed to execute our image semantic segmentation work. The model combines effective strategies to speed up the inference speed without sacrificing performance, which is based on different multi-scale features [23]. Originally, it performs downsampling operations on input images of various sizes. After a low-resolution image passes through the entire network, a rough prediction map is obtained. Compared with high-resolution predictions, it lacks dozens of tiny but valued details, and the boundaries of objects also become blurry [24,25]. Accordingly, the low-resolution boundary prediction can be optimized, and then additional loss is added to the segmentation boundary of the network output, which is simple and effective for the network to learn the edge and regional features of the target object.

Therefore, we realize that complex network depth information, large datasets, and real-time performance are all problems that need to be solved urgently in the application of autonomous driving technology. The development and popularization of semantic segmentation methods are still facing plenty of difficulties and challenges. First, autonomous driving has very strict requirements for computer vision and needs to meet real-time characteristics. Second, because its application involves driving safety issues, a certain accuracy of the semantic segmentation model must be guaranteed. In order to address these problems, we propose an improved lightweight real-time semantic segmentation network, using multi-scale branches and a cascaded feature fusion unit to extract rich multi-level features. In this paper, a spatial information network is designed to transmit more prior knowledge of spatial location and edge information. During the course of the training phase, we append an additional loss function to enhance the learning process of the deep learning network system as well. The model we propose can detect the road in the movable area from images and avoid obstacles such as pedestrians and vehicles, which not only increases the safety and comfort of car driving, but also meets the needs of assisted driving.

In summary, the main contributions of our work are threefold:

1. For road detection tasks, we propose a real-time semantic segmentation architecture, which enhances the image cascading network (ICNet) architecture based on real-time image semantic segmentation to deliver more spatial position prior knowledge and edge information.
2. We take the spatial information inference module as a sub-network and effectively integrate it with the semantic segmentation network. Furthermore, an additional loss function is introduced for the SP-ICNet architecture to enhance the learning process of the deep learning network system.
3. Contrary to the dramatic development of high-quality semantic segmentation, we focus on a more lightweight network to undertake semantic segmentation tasks and apply it to the public dataset to evaluate real performance, including the essential implementation details of road obstacle detection.

First, we introduce the detailed information of the model and method in Section 2. Then, we discuss the results in Section 3 and perform the corresponding analysis. Finally, conclusions are drawn in Section 4.

## 2. Methodology

**Figure 1:**
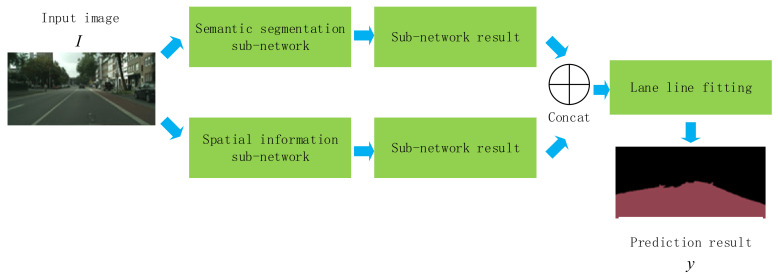
Overview of the performance with the proposed framework.

In this paper, a lightweight semantic segmentation algorithm for road obstacle detection is proposed, SP-ICNet, with the aim to extract the edge features in road images accurately and preserve the image boundary details by adding the spatial information sub-network of the original ICNet. Apart from this, an external loss is appended to the training stage of the spatial information sub-network output to ameliorate the learning ability of the model. Figure 1 shows an overview of the workflow of this study.

### 2.1. Semantic Segmentation Model

**Figure 2:**
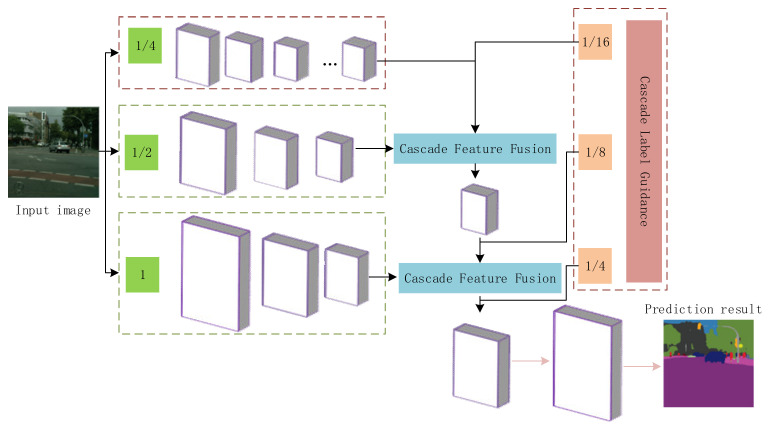
The workflow of the image cascade network.

In order to satisfy the real-time requirements, we adopt the ICNet model as a backbone semantic segmentation network to detect road obstacles. ICNet is a lightweight semantic segmentation network with fast detection speed and low memory consumption, which is consistent with the characteristics of strict real-time requirements and low hardware conditions in road obstacle detection [26]. As a state-of-the-art method, it introduces a cascaded feature fusion module on the basis of PSPNet, which dramatically combines the processing efficiency of low-resolution images and the detection accuracy of high-resolution images, maintaining a high balance between detection accuracy and detection speed. Its structure is shown in Figure 2, the operations are indicated in brackets, and the final × 4 upsampling is only used during testing.

In order to accelerate the speed of network segmentation, ICNet converts the input image to different scales and then inputs three branches: low-resolution, medium-resolution, and high-resolution images. Then, the pyramid pooling module (PPM), which can obtain global information capabilities, is retained, and the fused features after pyramid pooling are upsampled and output features. Further, it innovatively proposes the cascaded feature fusion (CFF) unit and the training method of cascaded label guidance, using these different levels of feature fusion and cascaded label guidance to produce a better prediction output and obtain the final segmentation.

#### 2.1.1. Branches of Different Scales 

The input image resolution of the low-resolution branch is only 1/4 of the original input image, aiming to extract the semantic information of the entire image, so it adopts the heavy CNN structure. As shown in Figure 2, after multiple downsamplings of convolutional layers, the resulting feature map size is 1/32 of the original input image. Then, it uses dilated convolution to expand the receptive field of the feature map without changing the size of the feature map. Similar to the operation of the low-resolution branch, after the medium-resolution (1/2) branch is downsampled and convolved, the resulting feature map size is 1/16 of the original image. Meanwhile, the two branches share the same parameters to increase the calculation speed. The high-resolution branch outputs a feature map of 1/8 the size of the original image after passing through three convolutional layers. Although the resolution of the input image is higher, the speed is faster due to fewer convolutional layers. The light CNN structure is adopted for medium resolution and high resolution.

#### 2.1.2. Pyramid Pooling Module 

This model uses the pyramid pooling module proposed by PSPNet in the heavy CNN network, which can aggregate the context information of different regions, thereby improving the ability to obtain global information. Experiments suggest that the model shows excellent results on multiple different datasets. First, it divides the feature map into different sub-regions by using an operation called adaptive average pooling. Then, the low-dimensional feature map is upsampled to obtain the same size features as the original feature map through bilinear interpolation. Finally, the features of different scales are summarized into the final global feature of the pyramid pool.

#### 2.1.3. Cascaded Feature Fusion Unit 

**Figure 3:**
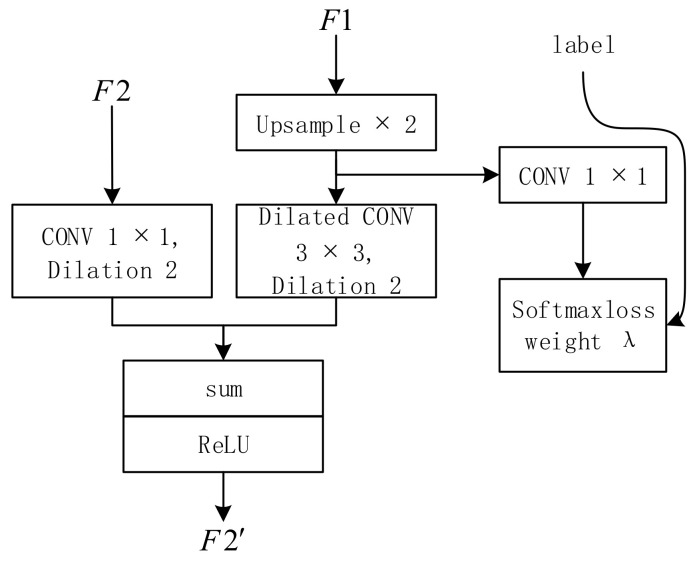
Cascaded feature fusion unit.

In order to combine the feature maps of different resolutions output by the three branches, ICNet proposes a cascaded feature fusion unit. Since the output feature map size ratio between the three branches is fixed at 2, the CFF upsamples the smaller feature maps of the two input feature maps twice, and then uses a convolution kernel size of 3 × 3 dilated convolution layers to expand the receptive field of the feature map and keep the resolution of the feature map unchanged. This operation makes the resolution of the two input feature maps of the CFF unit the same and then adds the two feature maps to the ReLu layer. At the same time, in order to enhance the learning of F1, auxiliary labels are applied on the upsampling features of F1. Its structure is shown in Figure 3.

#### 2.1.4. Cascading Label Guidance Strategy 

For reinforcing the learning of features in the three branches, the network adopts the loss function optimization strategy, which is described in PSPNet, and adds a cascaded label guidance strategy to the CFF. The specific method is to double the smaller input size in the CFF unit sample and use dilated convolution to broaden the receptive field and different scales (such as 1/16, 1/8, and 1/4) of ground truth labels to guide the learning stage of low-, medium-, and high-resolution inputs. Among them, the high-resolution branch does not need to be cascaded with the higher-resolution feature map, so when calculating the loss, the 1/4-size label map of the original image corresponds to the feature map in the decoding stage after the three branches are fused.

On the basis of the ICNet network, we construct a parallel spatial information sub-network to extract and conserve the road edge detail information and then merge it with the features extracted by the original network. Meanwhile, the SLIC superpixels would be generated as an auxiliary training branch in another sub-network, which is also appended to an external loss. The richer feature map space representation improves the performance of the proposed model in learning detailed features and obtains the final semantic segmentation results precisely.

### 2.2. Spatial Information Sub-Network

The ICNet network we adopted in this work is to let low-resolution images pass through a complete heavy network first. Then, according to a novel strategy, the medium- and high-resolution features are merged, so that the network has both the advantages of accuracy and speed, which is challenging for segmentation networks. However, in the traditional method, all pixels are involved in the calculation and classified into specific categories for scene parsing, which is difficult for segmentation networks. For its constraints by pixel information and spatial unity, while the highly computational complexity also cannot satisfy expectations, it will affect the high-quality segmentation of road images.

Instead, inspired by the image edge information construction network [27,28,29], while using pixels for semantic segmentation, we construct a spatial information sub-network. The superpixels generated by the SLIC method will participate in this model, and they combine the spatial context information of the superpixels and fuse high-level abstract semantic features to acquire semantic segmentation results with the edge optimized. It solves the problem that a large amount of image detail information is lost due to continuous pooling and downsampling in the feature extraction stage of the existing deep learning-based image semantic segmentation algorithm, which lose some important edge information of the object. Meanwhile, superpixels are especially widely used in traditional energy minimization frameworks, as they can extremely promote the performance of the algorithm without increasing the computational complexity.

**Figure 4:**
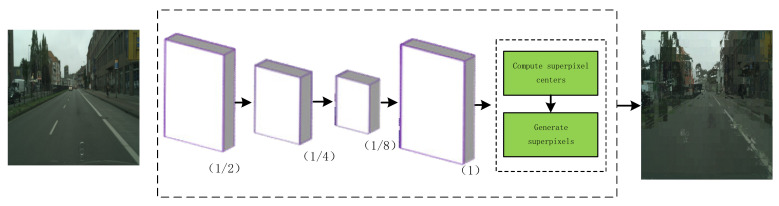
Spatial information sub-network.

The detailed structure of the spatial information sub-network is shown in Figure 4. In the spatial information detection sub-network, the input image first generates features at each pixel through the sub-network. The entire network is divided into five stages: First, there are three convolutional layers in the spatial information sub-network. The input image is downsampled by factors of 2 and 4, which will output a feature map of 1/8 the size of the original image after convolution, and uses dilated convolution to expand the receptive field of the feature map. This can avoid the loss of high-level feature information in operations such as pooling and capture the edge information of the target better. Then, through deconvolution, the feature map is upsampled to the original image size resolution. In the next stage, combining these network features, the SLIC iterative clustering is performed to obtain the superpixels segmentation results. The algorithm restricts the image search space to an area proportional to the size of the superpixel, and then calculates the center of the superpixel cluster, using the weighted combination of color and space metrics as the distance measurement unit between each pixel and the cluster center to generate a superpixel block with a regular and compact size. Finally, the result of the spatial information sub-network segmentation has obvious boundary discrimination, which helps to obtain more edge information of the target and maintain the integrity and clarity of the boundary.

A good SLIC algorithm is similar to a clustering algorithm, through the process of local clustering of image pixels, using color distance and spatial distance to search for pixel points and cluster centers. The generated superpixel blocks are relatively compact, similar in size, and have similar texture, brightness, and other pixel information, so that the neighborhood features are relatively well maintained, and the algorithm can better capture edge information. In the spatial information sub-network, the road image extracts rich local feature information after convolution and deconvolution operations, which helps SLIC generate better superpixel blocks, cascade the fusion of different feature information, merge this channel information, and obtain the segmentation results of the spatial information sub-network. Certainly, the image boundary information contained in these segmentation results helps to make up for the lack of boundary information of the basic network ICNet and improve the accuracy of the overall framework.

After the spatial information sub-network obtains the edge information, and the semantic segmentation sub-network obtains the semantic information, the concat feature fusion method is used to fuse the features from two branches. Owing to the spatial information sub-network and the semantic segmentation sub-network obtaining clearly different feature maps, the former represents more image edge and detail features, and the result obtained by the latter is more inclined to show the global regional features of the image. It means our framework will supply a richer feature expression to transmit details exactly.

### 2.3. External Auxiliary Loss Function

The improved road obstacle detection model SP-ICNet includes two branches: a semantic segmentation network and a spatial information sub-network. The branch of the semantic segmentation network is based on the ICNet model. During the course of the process, the model carries out the prediction and the branch of the spatial information sub-network conducts detecting road obstacles as an auxiliary training technique simultaneously. Therefore, this paper adds an external loss function in an auxiliary manner to optimize the learning process in the training stage of the model. It is noteworthy that the auxiliary training of the sub-network is only used in the training phase to adjust the network parameters and optimize the model, thus increasing the detection consequences of the road edge in images.

In the semantic segmentation network of road scenes, due to the uneven distribution of pixels, this paper uses the cross-entropy loss function to obtain the gradient of the model to update the network parameter values. Since the ICNet model has three branches with different resolutions, it adds a weighted SoftMax cross-entropy loss in each branch with a relevant loss weight $\lambda_{t}$. Therefore, the loss function of the semantic segmentation network is defined as

(1) $$L_{1}=-\sum_{t=1}^{T}\lambda_{t}\frac{1}{Y_{t}X_{t}}\sum_{y=1}^{Y_{t}}\sum_{x=1}^{X_{t}}\log\frac{e^{F_{i}{}^{t^{\prime }}}}{\sum_{j=1}^{N}e^{F_{j}{}^{t}}}$$

where T represents branches and N represents categories. The size of the feature map $F^{t}$ in each branch is $Y_{t}X_{t}$. The corresponding ground truth label of the feature map is $F^{t^{\prime }}$. Moreover, the loss weights $\lambda_{t}$ are set for each branch, which are 0.16, 0.4, and 1, respectively.

The loss function of the spatial information sub-network can be defined as

(2) $$L_{2}=-\frac{1}{M}\sum_{s=1}^{S}\sum_{i=1}^{N}y_{i}\log\frac{e^{z_{i}}}{\sum_{j=1}^{N}e^{z_{j}}}$$

where M represents samples, S represents superpixels, and N represents categories. The ground truth label of each pixel i is $y_{i}$.

Specifically, the total loss L of the improved model is acquired by the weighted summation of the semantic segmentation network loss and the spatial information sub-network loss. The semantic segmentation network is applied to detect road obstacles in the road scene images. In order to collect more potential semantic classification information and regional features precisely, we assign its weight to 1 in the total loss. The spatial information sub-network branch mainly assists the target classification on the basis of the semantic segmentation network and adjusts the network parameters, which relieves the problem of the semantic segmentation network in the road edge coarse segmentation to a certain extent. Through several sets of experiments, it is found that setting its weight to 1/10 of the semantic segmentation network can speed up the model convergence.

## 3. Results and Analysis

### 3.1. Dataset and Evaluation Metrics for Segment Model

Image segmentation methods based on the deep learning model often require a lot of data for training, and KITTI [30,31,32] and Cityscapes [33] are two famous and influential datasets for scene understanding and road analysis. They have contributed to the development of computer vision in the field of autonomous driving. Since the core idea of this article is to construct the entire architecture through the pixel semantic segmentation method to complete the task of road obstacle detection, the proposed SP-ICNet model is also evaluated on the Cityscapes dataset, which is the main benchmark for autonomous driving or environmental perception tasks. It is often used in scene parsing of urban roads to evaluate the performance of algorithms, such as semantic segmentation and instance segmentation. The Cityscapes dataset records various weather conditions, driving times, and road environments in 50 cities, which cover almost all the conditions of vehicles in daily driving. Moreover, the resolution of the Cityscapes dataset is relatively high. The resolution of all images in the entire dataset is 2048 × 1024 pixels, providing 5000 high-quality pixel-level annotation images, 20,000 coarsely annotation images, and 30 annotated objects.

The accuracy analysis is crucial to measure the quality of semantic segmentation algorithms. To assess the performance of the proposed sub-network, we adopt three metrics for road object detection as follows [34]. They are defined as

(3) $$precision(P)=\frac{TP}{TP+FP}$$

(4) $$\mathrm{accuracy}(A)=\frac{TP+TN}{TP+FP+TN+FN}$$

(5) $$\mathrm{IoU}=\frac{TP}{TN+FP+FN}$$

Assume *TP* represents the true positives, *FP* is the false positives, *FN* is the false negatives, and *TN* is the true negatives. We use the popular evaluation metric mean Intersection over Union (mIoU), which is often used to evaluate the accuracy of semantic segmentation, and it simply takes the averages of IoU for whole classes of images.

### 3.2. Details of the Experiments

For the semantic segmentation sub-network, the ICNet classification network is adopted as a backbone of our model. It can quickly extract features of different scales, and we add a spatial information network to extract more position information of edge pixels simultaneously. Then, the prediction results of the two sub-networks are merged with a concat technique. All the works above are used to capture image information of different characteristics in each network as much as possible, for the purpose of aggregating global context information.

We implement our network using the TensorFlow framework and NumPy and input training images with a resolution of 720 × 720 pixels. In the semantic segmentation network, the weights for the Cityscapes are initialized using the pre-trained ICNet model, and we perform little fine-tuning on it. It uses a poly strategy for training optimization, the learning rate is set to 0.0001, the batch size is set to 4, and the number of iterations is 10,000. The momentum and weight decay are set to 0.9 and 0.0001, respectively. The spatial information sub-network is a simple architecture completely designed from scratch, without loading any pre-trained model weights. The size of the training image is 720 × 720 pixels, the batch size is set to 8, and other parameters remain the same. Certainly, the parameters of weights in the total loss function are set depending on the training data. In order to accelerate the model convergence, through a lot of experiments about the total loss, we set the weight of the semantic segmentation network loss to 1 and the weight of the spatial information sub-network to 1/10 of it.

### 3.3. Inference Run-Time Performance/Comparison of Segmentation Performance

**Figure 5:**
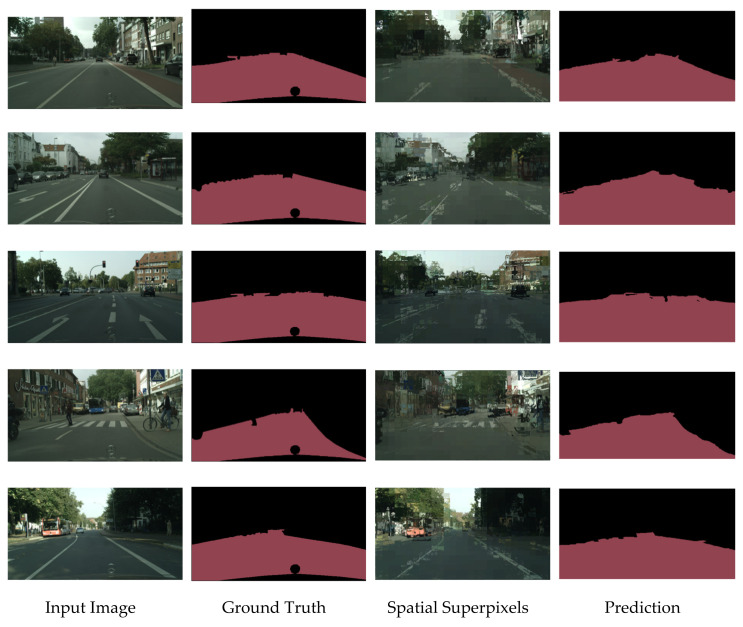
Segmentation results: evaluation results on Cityscapes validation set. From left to right are original RGB image, ground truth annotation, spatial superpixels, and prediction of our model.

In order to ascertain the effectiveness of the proposed image semantic segmentation model in the field of intelligent vehicle environment perception, we conduct multiple sets of experiments to assess and verify the visual segmentation effect and detection performance of the proposed model. Several popular semantic segmentation models and ICNet semantic segmentation models without spatial information network modules are compared with the results of the models proposed in this paper to analyze the performance of the algorithm. For this work, we mainly focus on segmentation and extraction of the drivable region and obstacles in front of the vehicle in the road image of the Cityscapes dataset. Some samples of the image semantic segmentation results of the proposed model in the Cityscapes dataset are shown in Figure 5.

In Figure 5, the first column is the input image, from the validation set of the Cityscapes dataset. The second column is the ground truth of road detection. The third column is the superpixel block generated by the spatial information sub-network. After fusion with the semantic segmentation network, the final predicted image is obtained, which is displayed in the fourth column. According to these samples, the proposed SP-ICNet in this paper shows good results that acquire an accurate segmentation of the road region, which is very approximately close to the ground truth label. The superpixels generated by the spatial information sub-network act as an auxiliary sub-network to make sure the edge information of the road region and obstacles are precisely detected, and the road segmentation results are relatively smooth and clear. This demonstrates that the proposed model does not easily make dangerous false alarms, for example, sidewalks and vehicles being judged as drivable road regions, which also guarantees the safety of vehicle driving to a certain extent.

The mIoU results of the experimental segmentation are shown in Table 1. In addition, we select several representative algorithms with higher correlations from the semantic segmentation network methods for comparison and describe their key technologies. Among these well-known algorithms, the FCN [35], DeepLab [36], and PSPNet [23] algorithms are more suitable for semantic segmentation of conventional static images. It is found that the mIoU of DeepLab and FCN is about 60%, and PSPNet exceeds 80% in particular, suggesting that it aggregates features of different scales in the image and performs with a good detection effect. Its good performance also implies that the PSPNet used as a heavy network for rough segmentation in our work is sensible. On the other hand, SegNet [37], ICNet [14], and the algorithm proposed in this paper are mainly used to carry out the test tasks of real-time image semantic segmentation in automatic driving. Their mIoU segmentation results exceed 50%, which basically satisfies the semantic segmentation requirements of obstacle detection in road scenes. It is substantial to notice that the algorithm in this paper adopted a cascaded feature network to fuse feature maps of different scales, capturing rich boundary features by a superpixel method in an auxiliary manner, which is significantly improved compared to others.

In addition, we also contrast the run-time of the algorithms, which are all performed on the Cityscapes dataset. It is necessary to note that in order to test the execution ability of the semantic segmentation network on non-GPU platforms, all training and validation works in this paper are carried out in the CPU environment. The running times of ICNet and the proposed algorithm shown in Table 2 are both thoroughly subjected to the real-time requirements. In fact, PSPNet shows excellent ability in algorithm accuracy. However, due to the consideration of extracting global information with different scales, its execution time is greater than 100 s, which simply means high precision with low speed. Nevertheless, there is no doubt that it is still a very compelling semantic segmentation network algorithm according to nowadays standards. Back to our experiment, we can also find that the segmentation run-time of the proposed algorithm is slightly longer than that of the ICNet algorithm, which is 0.79 s longer than the original one. This is because we have added a spatial information sub-network for auxiliary prediction. In general, these observations collectively demonstrate the effectiveness of this algorithm in terms of run-time and segmentation accuracy, which suggests that it is complete for real-time road image obstacle detection.

Again, in order to carry out further experimentation, we conduct several other sets of comparisons to assess whether the superpixels generated by the spatial information sub-network can help the algorithm enhance the boundary extraction of road images. We adopt three different evaluation criteria to verify it, in terms of accuracy, precision, and recall, respectively, as shown in Table 3. It can be seen that superpixels generated by the spatial information sub-network have substantial influences on the conservation of road edge information. Obviously, the proposed SP-ICNet has achieved 85.4% accuracy, 84.3% precision, and 83.9% recall on Cityscapes. The proposed model shows advantages in terms of different metrics, which are the tasks undertaken to obtain details of the edge information of roads and obstacles.

**Figure 6:**
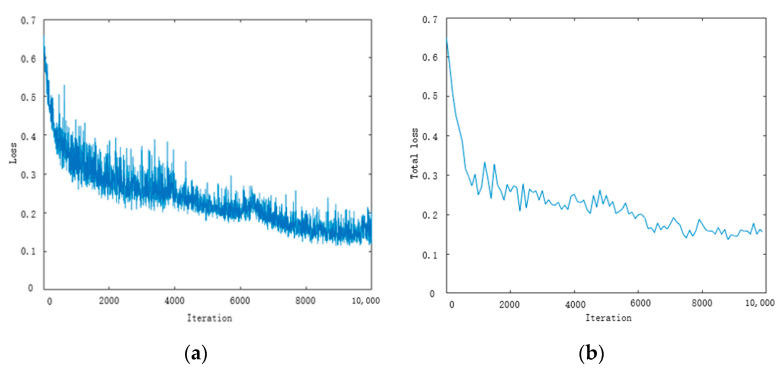
The loss plot for the semantic segmentation model and the total loss with an external auxiliary network in the training stage.

Next, we use the weight of the pre-trained model (ICNet pre-trained on the Cityscapes dataset) to initialize the weight of the model proposed in this article on the training set. While the prediction is carried out at the training stage, we extend an additional loss function as an auxiliary training to adjust the network parameters and optimize the network model, improving the network’s detection effect on road edges. In Figure 6, the left image shows the loss of the semantic segmentation network, and the right image shows the total loss of the entire segmentation framework. It can be seen that the total loss in the training process decreases after 8k iterations and nearly tends to stabilize. The segmentation framework can converge well, which indicates that our model is reliable.

## 4. Conclusions

In this paper, an SP-ICNet model has been proposed, which uses an additional sub-network to acquire richer feature information of the road images to achieve drivable region and obstacle detection naturally. A semantic segmentation network, ICNet, based on feature fusion is adopted in our work. By capturing contextual information, we avoid the problems of large computation and memory consumption caused by using probability graph models. The methods of the pyramid pooling module, multi-scale convolution, and the cascade model are used to fuse feature information of different scales to gradually refine the segmentation results. As a state-of-the-art lightweight network, it has absolute advantages in predicting speed. However, along with its advantages, it also has disadvantages such as the boundary information of the segmentation target not being detected completely. Therefore, this paper uses the superpixels method as the spatial information sub-network, which utilizes the local and global feature information of the image thoroughly to merge edge features and semantic segmentation features, improving the problem of fuzzy edges and inaccurate segmentation in semantic segmentation. The experimental results demonstrate the effectiveness of this model, and we conduct multiple sets of experiments to assess and verify the visual segmentation effect and detection performance of the proposed model. Although there are some inescapable errors in the displayed road detection results, in general, it can obtain more ideal semantic segmentation results, and it acquires the drivable area and obstacles in road images of popular datasets, which is significantly improved compared to other models. Moreover, it meets the test task of real-time image semantic segmentation requirements in autonomous driving, and the entire network architecture can converge very well. The model is relatively stable and reliable, which indicates that the proposed model in this paper is a beneficial attempt and contributes to the research on image semantic segmentation to a certain extent.

Real-time image semantic segmentation is often used in environment perception tasks such as scene analysis and multi-target detection, where it has great application value. Furthermore, we plan to enhance the ability of our semantic segmentation network in more possible dimensions and study the detection of different targets in the road scene without sacrificing high-quality performance. Similarly, it will be interesting to explore ways to achieve safer driving on road corner subjects or seek compression models to achieve real-time requirements for autonomous driving more efficiently. These are interesting research directions.

## Figures and Tables

**Table 1:** Performance comparison of the proposed algorithm and other algorithms on the Cityscapes dataset.

Algorithm	Backbone	Key Technology	mIoU (%)
FCN-8s [35]	VGG16	Upsampling, Skip layer	65.3
DeepLab [36]	ResNet	Upsampling, Structure prediction	63.1
SegNet [37]	VGG16	Deconvolution, Upsampling, DropOut layer	57.0
PSPNet [23]	ResNet	Multi-scale feature integration, Spatial pyramid pooling	81.2
ICNet [14]	ResNet	Cascade model, Multi-scale feature integration	70.5
Ours	ResNet	Cascade model, Spatial information module	72.2

**Table 2:** Predicted inference time and frame on Cityscapes test set.

Algorithm	CPU Time (s)	Frame (fps)
ICNet	2.05	0.47
Ours	2.84	0.51

**Table 3:** Comparison of road region and obstacle segmentation accuracy (in %) using different metrics.

Algorithm	Accuracy	Precision	Recall
ICNet	82.7	80.6	81.6
Ours	85.4	84.3	83.9
